# IL-17 Attenuates Degradation of ARE-mRNAs by Changing the Cooperation between AU-Binding Proteins and microRNA16

**DOI:** 10.1371/journal.pgen.1003747

**Published:** 2013-09-26

**Authors:** Saheli Chowdhury, Annemiek Dijkhuis, Sabrina Steiert, René Lutter

**Affiliations:** 1 Department of Respiratory Medicine, Academic Medical Centre, University of Amsterdam, Amsterdam, The Netherlands; 2 Department of Experimental Immunology, Academic Medical Center, University of Amsterdam, Amsterdam, The Netherlands; Katholieke Universiteit Leuven, Belgium

## Abstract

Interleukin 17A (IL-17), a mediator implicated in chronic and severe inflammatory diseases, enhances the production of pro-inflammatory mediators by attenuating decay of the encoding mRNAs. The decay of many of these mRNAs depends on proteins (AUBps) that target AU-rich elements in the 3′-untranslated region of mRNAs and facilitate either mRNA decay or stabilization. Here we show that AUBps and the target mRNA assemble in a novel ribonucleoprotein complex in the presence of microRNA16 (miR16), which leads to the degradation of the target mRNA. Notably, IL-17 attenuates miR16 expression and promotes the binding of stabilizing AUBps over that of destabilizing AUBps, reducing mRNA decay. These findings indicate that miR16 independently of a seed sequence, directs the competition between degrading and stabilizing AUBps for target mRNAs. Since AUBps affect expression of about 8% of the human transcriptome and miR16 is ubiquitously expressed, IL-17 may in addition to inflammation affect many other cellular processes.

## Author Summary

Inflammation is driven by inflammatory mediators. Interleukin 17A (IL-17) is implicated in chronic and severe inflammation and exaggerates production of inflammatory mediators. This is due, at least in part, to the IL-17-attenuated degradation of mRNAs encoding these inflammatory mediators, but the underlying mechanism has remained elusive. Most of these mRNAs contain AU-rich elements in their 3′-untranslated region and are targeted by AU-binding proteins (AUBps) that promote either mRNA degradation or stabilization. Here we show that IL-17 directs the AU-mediated mRNA degradation (AMD pathway) by modulating the interaction of degrading and stabilizing AUBps via microRNA16 (miR16). Whereas microRNAs target mRNAs to the RISC pathway for degradation by binding to a seed sequence, miR16 drives degradation by the AMD pathway without an apparent seed sequence. Transcriptome analyses have revealed that the expression of 8% of all eukaryotic transcripts is dependent on the AMD pathway. Therefore, the impact of IL-17 on inflammatory diseases may extend beyond the production of inflammatory mediators to processes like tissue repair, cell cycle, etc. In addition, targeting AUBp and/or miR16 may provide a novel therapeutic option to combat the IL-17 axis of inflammation.

## Introduction

Interleukin-17A (IL-17), the prototype of the IL-17 cytokine family [1], [2], is a major player in the development of auto-immunity, host defence and inflammation [3]. IL-17 acts as a homodimeric molecule, although it can form heterodimers with IL-17F [2], and predominantly activates stromal cells via the receptors IL-17RA and IL-17RC [4]. IL-17 by itself, but most markedly in synergy with pro-inflammatory stimuli such as TNF-α, controls the production of pro-inflammatory mediators. IL-17 has been shown to induce gene transcription via the NFκB pathway by up-regulating IκBζ [5] and that of C/EBP by promoting transcription of C/EBP-β and C/EBP-δ [6]. IL-17 has also been shown to attenuate mRNA degradation via MAPK [7] and IKKi [8]. These transcriptional and post-transcriptional mechanisms may underlie the enhanced production of inflammatory mediators by IL-17. For lung epithelial (-like) cells we have established previously that IL-17 synergizes with the TNF-α-induced production of the pro-inflammatory mediators IL-8 and IL-6 by attenuating degradation of the respective mRNAs [9], with a weak transcriptional effect at most. The mechanism by which IL-17 attenuates mRNA degradation has remained elusive.

A major mRNA decay pathway is mediated by AU-rich-elements (AREs) in the 3′-untranslated region (3′-UTR) of mRNAs [10]. These AREs are found in 8% of the human transcripts encoding inflammatory mediators, transcription factors, proteins controlling the cell cycle, and many other classes of proteins, affecting virtually all cellular and physiological processes [11]. Although the machinery involved in this ARE-mediated mRNA decay (AMD) pathway is still far from clear, over the last 10–15 years a family of proteins has been discovered that recognizes these AREs and influence AMD. The proteins tristetraprolin (TTP) [12], AU-binding factor 1 (AUF-1) [13], the ELAV-like protein HuR [14], [15], KH-type splicing regulatory protein (KHSRP) [16], T-cell intracellular antigen (TIA)-1 [17] and TIA-1-related protein (TIAR) [18] are all acknowledged ARE-binding proteins. TTP, AUF-1 and KHSRP have been shown to promote mRNA degradation, HuR and also AUF-1 was shown to attenuate mRNA degradation, whereas TIA-1 and TIAR mediate translational repression. The prevailing paradigm is that the balance of the various AUBps determines the effect on the targeted mRNA [19].

More recently, small non-coding RNA molecules, called microRNAs (miRs), have been implicated in 3′-UTR-mediated mRNA degradation [20]. miRs bind a target mRNA via a seed sequence and, for animal cells, complementarity between the miR and the target mRNA promotes mRNA degradation [20]. This degradation is facilitated by catalytic argonaute proteins in the RISC complex [21].

We set out to delineate the mechanism by which the degradation of mRNAs encoding for IL-8, GM-CSF and IL-6 is regulated. These mRNAs assembled with various AUBps in a novel ribonucleoprotein complex in the presence of miR16, which lead to their decay. For example, IL-8 mRNA degradation was cooperatively promoted by TTP, KHSRP and miR16. Alternatively, IL-8 mRNA degradation was inhibited by AUF-1 in the absence of miR16. IL-17 attenuated miR16 expression and binding to the complex, and markedly promoted binding of IL-8 mRNA to AUF-1, in line with stabilization of IL-8 mRNA. Similar findings were obtained for other ARE-containing mRNAs, indicating that the various AUBps compete for binding to the target mRNA, the balance of which is regulated by IL-17, likely via miR16. This suggests that miR16 may direct competition between the AUBps and herewith the expression of proteins encoded by ARE-containing transcripts, many of which have regulatory functions that contribute to the pathophysiology of chronic inflammatory diseases [11].

## Results

### TTP and KHSRP limit IL-8 protein production and mediate degradation of IL-8 mRNA

**Figure 1:**
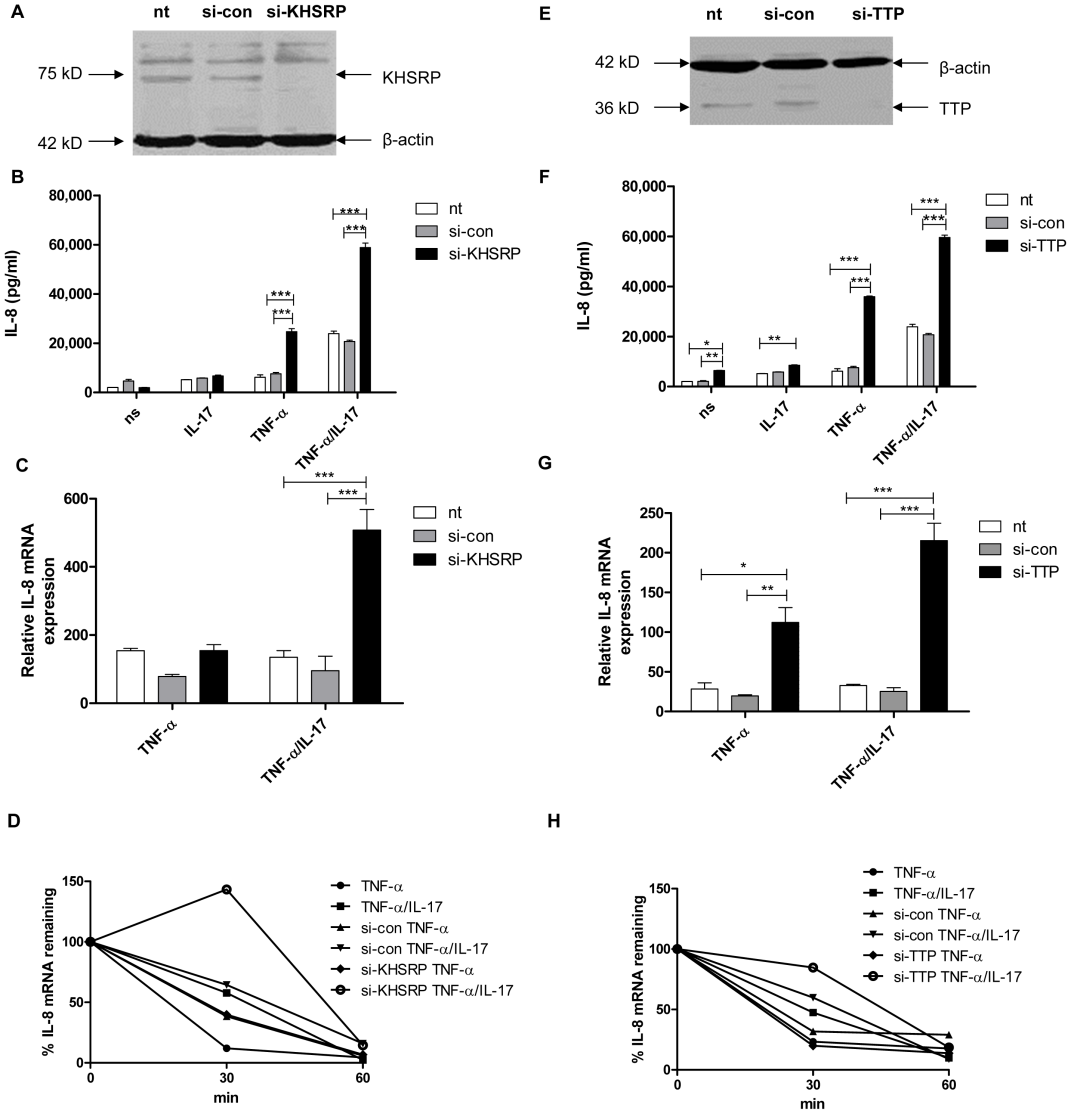
KHSRP and TTP down-regulate IL-8 expression by promoting degradation of IL-8 mRNA. **a, e**). KHSRP (75 kD) (**a**) and TTP (36 kD) (**e**) were knocked down in NCI-H292 cells using si-RNA. Scrambled si-RNA (si-con) served as a control for specificity and β-actin (42 kD) was determined to verify equal protein loading. **b, f**). IL-8 released over 24 h in supernatant from NCI-H292 cells, non-transfected (nt) or transfected with si-con or si-KHSRP (**b**) or si-TTP (**f**), and either non-stimulated (ns) or stimulated with IL-17 (100 ng/ml), TNF-α (5 ng/ml) or TNF-α plus IL-17 (5 ng/ml+100 ng/ml, respectively). Data are shown as mean ± SD. 2-way ANOVA analysis was done with Bonferroni's multiple comparison post-test. ***P<0.001, **P<0.01, *P<0.05. **c, g**). Quantitative PCR (q-PCR) analysis of IL-8 mRNA was done for non-transfected or transfected cells with si-con or si-KHSRP (**c**) or si-TTP (**g**), at 2 h after stimulation with TNF-α or TNF-α plus IL-17, or left unstimulated. ***P<0.001, **P<0.01, *P<0.05 (2-way ANOVA, Bonferroni's multiple comparison post-test). The data presented is representative of 3 independent experiments done for each AUBp. mRNA results are presented relative to that of unstimulated, non-transfected cells. **d, h**). Non-transfected, si-con or si-KHSRP (**d**) or si-TTP (**h**) transfected cells were left unstimulated or stimulated with TNF-α or TNF-α plus IL-17 for 2 h. RNA was isolated from cells at 0, 30 and 60 minutes (mins) after blocking gene transcription by actinomycin D (5 µg/ml). % of IL-8 mRNA remaining was calculated on basis of q-PCR. Representative experiment is shown representative of 5 experiments.

Previously we have shown that IL-17 synergizes with TNF-α in IL-8 protein production by human lung epithelial-like NCI-H292 cells as well as by human primary bronchial epithelial cells, primarily by attenuation of IL-8 mRNA degradation [9]. The AUBps KHSRP, TTP and AUF-1 have been implicated in IL-8 mRNA degradation [22]–[27] in line with the presence of nine AUUUA repeats, four of which are clustered in the 3′-UTR of IL-8 mRNA [28], [29]. To delineate the mechanism by which IL-17 inhibits IL-8 mRNA degradation we first assessed the contribution of the AUBps KHSRP and TTP to IL-8 production by NCI-H292 cells. KHSRP and TTP are expressed by NCI-H292 cells and were effectively knocked down using specific si-RNAs (Fig. 1a and e). Cells in which KHSRP or TTP was knocked down showed markedly higher IL-8 protein production over 24 h of stimulation (Fig. 1b and f) in cells stimulated with TNF-α plus IL-17 and, less so, in response to TNF-α. TTP, but not KHSRP, also enhanced IL-8 protein production in cells exposed solely to IL-17 or no stimulus, but as the overall effects were limited we focused on stimulation by TNF-α plus IL-17 versus TNF-α alone. IL-8 mRNA expression at 2 h after exposure to IL-17 plus TNF-α or TNF-α alone, i.e. when IL-8 mRNA expression is at its highest [9], was significantly higher in the absence of TTP (Fig. 1g). Knockdown of KHSRP also led to significantly enhanced IL-8 mRNA expression, but only in the presence of TNF-α plus IL-17 (Fig. 1c). So, both KHSRP and TTP attenuate IL-8 protein production by down regulating IL-8 mRNA expression in cells exposed to TNF-α plus IL-17. IL-17 enhanced the stability of IL-8 mRNA, as shown before [9]. Markedly, the absence of KHSRP or TTP further attenuated IL-8 mRNA degradation after stimulation by TNF-α plus IL-17 (Fig. 1d and h). This confirms that TTP and KHSRP promote degradation of IL-8 mRNA. We saw no attenuation of IL-8 mRNA degradation after 2 h of stimulation with TNF-α, which may relate to the rapid kinetics of IL-8 mRNA expression.

### AUF-1 attenuates degradation of IL-8 mRNA and enhances IL-8 protein production only in the presence of IL-17

**Figure 2:**
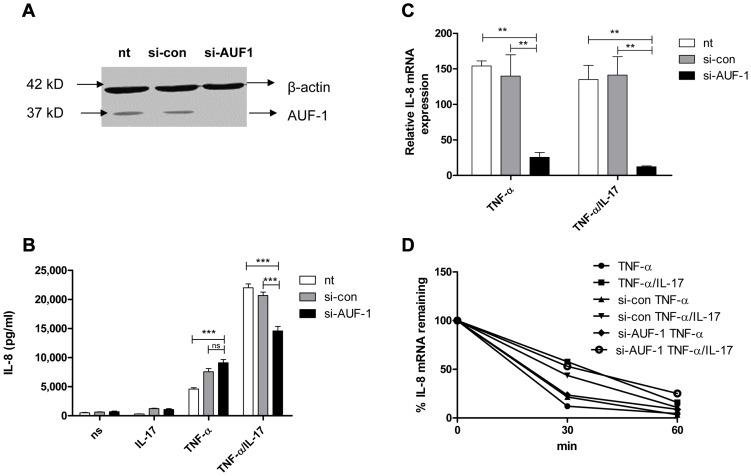
AUF-1 enhances IL-8 expression by halting IL-8 mRNA degradation. **a**) AUF-1 (approx. 37 kD) was knocked down in NCI-H292 cells with si-AUF-1. Scrambled si-RNA (si-con) served as a control for specificity and β-actin (42 kD) was determined to verify equal protein loading. **b**). IL-8 released over 24 h in supernatant from NCI-H292 cells, non-transfected (nt) or transfected with si-con or si-AUF-1 and either non-stimulated (ns) or stimulated with IL-17, TNF-α or TNF-α plus IL-17. Data are shown as mean ± SD. ***P<0.001 (2-way ANOVA, Bonferroni's multiple comparison post-test). **c**). Q-PCR analysis of IL-8 mRNA was done for non-transfected or transfected cells with si-con or si-AUF-1 at 2 h after stimulation with TNF-α or TNF-α plus IL-17, or left unstimulated. **P<0.01 (2-way ANOVA, Bonferroni's multiple comparison post-test). mRNA results are presented relative to that of unstimulated, non-transfected cells **d**). Non-transfected, si-con or si-AUF-1 transfected cells were left unstimulated or stimulated with TNF-α or TNF-α plus IL-17 for 2 h. RNA was isolated from cells at 0, 30 and 60 mins after blocking gene transcription by actinomycin D. % of IL-8 mRNA remaining was calculated on basis of q-PCR. Representative experiments are shown for 3 experiments.

AUF-1, another major AUBp, has been proposed to halt IL-8 mRNA decay [25]. Efficient knockdown of AUF-1 in NCI-H292 cells (Fig. 2a) reduced IL-8 mRNA expression by 80–90% (Fig. 2c) in cells exposed to TNF-α plus IL-17 or to TNF-α alone. This indicates that AUF-1 indeed protects IL-8 mRNA from degradation. Knockdown of AUF-1 enhanced IL-8 protein production in response to TNF-α plus IL-17, but not to TNF-α alone (Fig. 2b). This suggests that IL-17 promotes the contribution of AUF-1 to IL-8 protein expression.

After stimulation by TNF-α or TNF-α plus IL-17, IL-8 mRNA decay was not affected by knockdown of AUF-1 (Fig. 2d). This was unexpected and may relate to very low IL-8 mRNA levels after si-AUF-1 (Fig. 2c), precluding accurate measurement of mRNA decay.

### miR16 limits IL-8 protein production and mediates degradation of IL-8 mRNA

**Figure 3:**
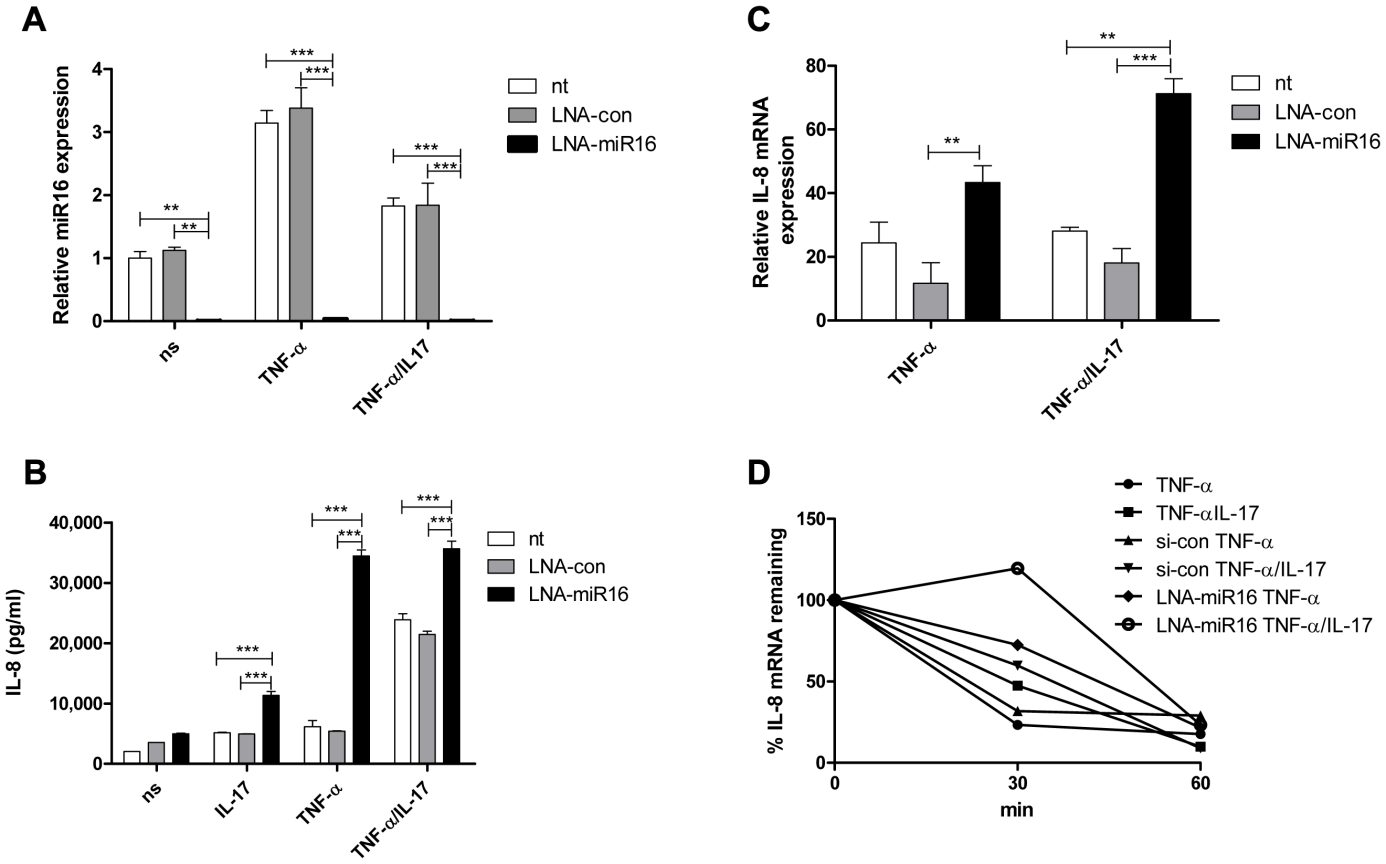
miR16 down-regulates IL-8 expression by promoting IL-8 mRNA degradation. **a**). Q-PCR showing the availability of miR16 was reduced in NCI-H292 cells using LNA-modified anti-miR16 and scrambled LNA (LNA-con) as compared to non-transfected (nt) cells whether non-stimulated (ns) or stimulated with TNF-α or TNF-α plus IL-17 for 2 h. miR16 is presented relative to that of unstimulated, non-transfected cells. ***P<0.001, **P<0.01 (2-way ANOVA, Bonferroni's multiple comparison post-test). **b**) IL-8 released over 24 h in supernatant from NCI-H292 cells, non-transfected (nt) or transfected with LNA-con or LNA-miR16 and either non-stimulated (ns) or stimulated with IL-17, TNF-α or TNF-α plus IL-17. ***P<0.001 (2-way ANOVA, Bonferroni's multiple comparison post-test). **c**). Q-PCR analysis of IL-8 mRNA was done for non-transfected or transfected cells with LNA-con or LNA-miR16 at 2 h after stimulation with TNF-α or TNF-α plus IL-17, or left unstimulated. ***P<0.001, **P<0.01 (2-way ANOVA, Bonferroni's multiple comparison post-test). mRNA results are presented relative to that of unstimulated, non-transfected cells. **d**). Non-transfected, LNA-con or LNA-miR16 transfected cells were left unstimulated or stimulated with TNF-α or TNF-α plus IL-17 for 2 h. RNA was isolated from cells at 0, 30 and 60 mins after blocking gene transcription by actinomycin D. % of IL-8 mRNA remaining was calculated on basis of q-PCR. Representative experiments are shown for 3 experiments.

A possible link between the miR pathway and the AMD pathway was reported by Han and co-workers [30]. To determine whether miR16 affects IL-8 production, cells were treated with a locked nucleic acid (LNA)-modified probe complementary to miR16 (anti-miR16) to limit the availability of miR16 (Fig. 3a). IL-8 protein production and IL-8 mRNA levels were enhanced markedly in anti-miR16-treated cells exposed to TNF-α or TNF-α plus IL-17 (Fig. 3b and c). IL-8 mRNA decay in these cells was decreased in the presence of TNF-α alone and of TNF-α plus IL-17 (Fig. 3d). Treatment with anti-miR16 also enhanced IL-8 protein production induced by IL-17 alone.

Collectively, these findings indicate that TNF-α- and TNF-α plus IL-17-induced IL-8 mRNA expression is downregulated by miR16, TTP and KHSRP, whereas AUF-1 enhances IL-8 mRNA expression. The relative contributions of each of the AUBps and miR16 vary depending on the presence or absence of IL-17.

### TTP, KHSRP and AUF-1 complex with IL-8 mRNA and miR16

It is unclear whether the AMD pathway and that directed by miR16 merge or are two independent pathways. In an initial attempt to address this, miR16 and IL-8 mRNA in cell lysates from TNF-α- or TNF-α plus IL-17-stimulated cells were labelled by ^32^P-probes and analyzed by RNA electrophoretic mobility shift assay (REMSA; Supporting Information Fig. S1). Although these analyses were difficult to accomplish and the resulting blots far from optimal, miR16 was found to co-migrate with IL-8 mRNA suggesting that they are in a large molecular weight complex. Co-incubation with an anti-TTP antibody super-shifted some IL-8 probe suggestive of a TTP-IL-8 mRNA complex in which TTP was accessible to anti-TTP. Failure to detect miR16 in this super-shifted material may indicate its absence, but may also be due to lack of sensitivity as the detection of miR16 by the labelled probe might be obscured by the antibody and vice-versa. Taken together this indicates that IL-8 mRNA and miR16 assemble in a complex with the AUBps. Interestingly, in the presence of TNF-α plus IL-17, a fraction of the IL-8 mRNA ended up in a faster moving band. In addition, in a competition assay with non-radioactive oligo probe against IL-8 mRNA, the slower migrating band was observed in the presence of TNF-α, while the faster migrating band was observed in the presence of TNF-α plus IL-17 (Supporting Information Fig. S2). This suggests that IL-17 changes the conformation of the complex, possibly related to an altered binding and cooperation between IL-8 mRNA, miR16 and the AUBps.

**Figure 4:**
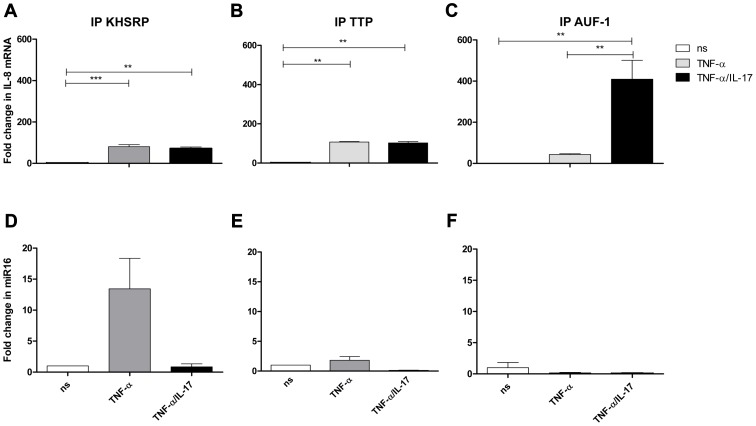
IL-8 mRNA and miR16 present in immuno-purified preparations of AUBps. **a–c-**) Fold change in IL-8 mRNA associated with immuno-purified KHSRP (**a**), TTP (**b**) and AUF1 (**c**) in NCI-H292 cells stimulated for 2 h with TNF-α or TNF-α plus IL-17 as compared to non-stimulated (ns) cells. Statistical analysis was done with 1-way ANOVA, Bonferroni's multiple comparison post-test. ***P<0.001, ** P<0.01. **d–f**) Fold change in miR16 associated with immuno-purified KHSRP (**d**), TTP (**e**) and AUF-1 (**f**) in NCI-H292 cells stimulated for 2 h with TNF-α or TNF-α plus IL-17 with respect to non-stimulated cells. Representative experiments are shown for 3 experiments.

To further determine whether IL-8 mRNA and miR16 interact with TTP, KHSRP and AUF-1, these AUBps were immuno-purified from cell lysates. To limit non-specific interactions the lysates were eluted on a column containing control antibodies prior to purification with the respective primary antibodies. Subsequently, the bound material was washed stringently with a low ionic strength buffer followed by a high ionic strength buffer before eluting the bound material under denaturing conditions. This resulted in an almost complete recovery of the targeted AUBps from the cell lysates as was visualized by Western blotting (Supporting Information Fig. S3). Both IL-8 mRNA and miR16 co-purified with these immuno-purified preparations (Fig. 4, explained further in next paragraph), which is suggestive of a core complex that binds various AUBps, miR16 and target mRNAs.

### IL-17 promotes binding of IL-8 mRNA to AUF-1 and limits that of miR16

Cell lysates from unstimulated cells and cells exposed to TNF-α, with or without IL-17, were prepared and the various AUBps were immuno-purified, as described above. Quantitative PCR analyses of IL-8 mRNA revealed an 80- and 100-fold increase of IL-8 mRNA bound to KHSRP and TTP, respectively, in lysates from cells exposed to TNF-α as compared to that of unstimulated cells (Fig. 4a and b). IL-8 mRNA bound to AUF-1 in TNF-α-exposed cells was 50-fold higher as compared to that in unstimulated cells (Fig. 4c). Interestingly, in lysates from cells exposed to TNF-α plus IL-17 *versus* those exposed to TNF-α only, IL-8 mRNA bound to AUF-1 significantly increased 10-fold, whereas that to KHSRP and TTP remained unchanged. As a control we also assessed IL-8 mRNA bound by isotype (IgG) control and found no changes in IL-8 mRNA for lysates from unstimulated cells, or cells exposed to TNF-α or to IL-17 plus TNF-α (Supporting Information Fig. S4a). This indicates that the binding of IL-8 mRNA to the various AUBps is specific.

Similar analyses for miR16 showed a 12-fold increase of miR16 with immuno-purified KHSRP (Fig. 4d), while only a 2-fold increase was observed in immuno-purified TTP from lysates of TNF-α-stimulated cells as compared to that of resting cells (Fig. 4e). Notably, co-exposure to IL-17 almost completely abolished miR16 in immuno-purified KHSRP and TTP. Another striking observation was the low miR16 presence in immuno-purified AUF-1 in lysates from cells exposed to TNF-α or TNF-α plus IL-17 (Fig. 4f). Although the differences in fold change for miR16 associated to the various AUBps under the three conditions tested were small, the differences between AUBps indicate that the association between miR16 and AUBps are specific interactions. The specificity of the binding of miR16 to the respective AUBps was also verified by analyzing material bound by the isotype-control IgG. No differences were observed with the various lysates (Supporting Information Fig. S4b).

This indicates that there is an intricate interplay between the various AUBps and miR16 in regulating IL-8 production, which also differs between cells exposed to TNF-α or TNF-α plus IL-17. Thus, in cells exposed to TNF-α, in which there is a profound IL-8 mRNA degradation, IL-8 mRNA is bound predominantly by KHSRP, TTP and miR16, which were shown to promote IL-8 mRNA degradation. In cells exposed to TNF-α plus IL-17 most IL-8 mRNA is bound to AUF-1 with no miR16 associated, which inhibits IL-8 mRNA degradation. So, IL-17 changes the relative contribution of the AUBps and miR16. The underlying mechanism by which IL-17 causes these changes is unknown. Therefore, we assessed whether IL-17 affects miR16 expression and that of the various AUBps.

### IL-17 limits miR16 expression and promotes cytoplasmic translocation of AUF-1

**Figure 5:**
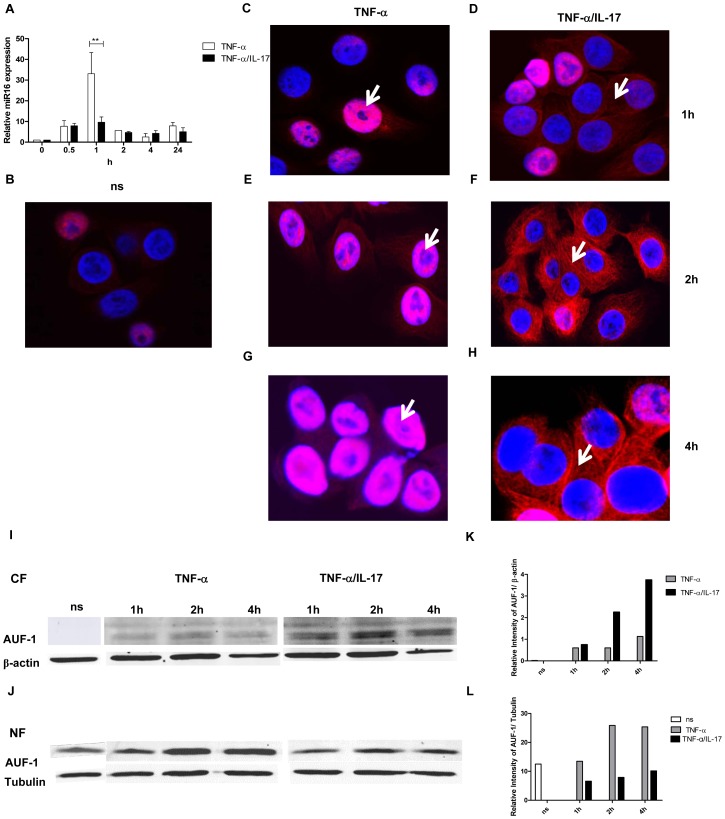
IL-17 limits miR16 expression and promotes cytoplasmic localization of AUF-1. **a**) Q-PCR showing time dependent expression of miR16 measured in NCI-H292 cells stimulated with TNF-α or TNF-α plus IL-17. miR16 is presented relative to that of unstimulated, non-transfected cells. **b–h**) Representative confocal images showing the localization of AUF-1 in resting NCI-H292 cells (**b**) and in cells stimulated with TNF-α (**c, e, g**) or TNF-α plus IL-17 (**d, f, h**) for 1, 2 and 4 h respectively as described in methods. The nucleus is stained with DAPI. White arrows indicate the predominant localization of AUF-1. Two experiments were performed with similar results. **i, j**) Western Blot analysis of AUF-1 in the cytoplasmic (**i**) and nuclear (**j**) fractions of resting cells (ns) or cells stimulated with TNF-α or TNF-α plus IL-17. β-actin and tubulin are markers for the cytoplasmic and nuclear fractions respectively. **k, l**) Quantitative analysis of AUF-1 normalised to β-actin in cytoplasmic fraction (**k**) and to tubulin in nuclear fraction (**l**) obtained from the Western Blot.

First, we determined miR16 expression by Q-PCR in response to TNF-α and TNF-α plus IL-17 over time. MiR16 was transiently induced with 30-fold increase at 1 h after stimulation by TNF-α (Fig. 5a), whereas co-exposure to IL-17 restricted the TNF-α-induced-miR16 expression to about 10-fold. This reduced miR16 expression is in line with attenuated IL-8 mRNA degradation, likely caused by limited miR16 availability in cells exposed to TNF-α plus IL-17.

Next, we assessed whether IL-17 affected the expression and cellular distribution of the various AUBps. Although the amounts of AUF-1, TTP and KHSRP over 24 h in total lysates from resting cells increased as compared to those exposed to TNF-α, we did not find marked differences in the amounts of these proteins between cells treated with TNF- α alone and TNF-α plus IL-17 (data not shown). We then looked at the nuclear and cytoplasmic expression of these proteins over time. There was some expression of AUF-1 in nuclei from resting cells (Fig. 5b). After stimulation with TNF-α for 1, 2 and 4 h AUF-1 expression was primarily restricted to nuclei (as indicated by white arrows) although at 2 and 4 h some cytoplasmic staining was detectable (Fig. 5c, e and g). Exposure to TNF-α plus IL-17 resulted in increased translocation of AUF-1 to the cytoplasm at 1 h, which was more pronounced at 2 and 4 h, with far less nuclear AUF-1, indicated by white arrows (Fig. 5d, f and h). This was confirmed by Western blotting where cytoplasmic AUF-1 was not observed in resting cells but it markedly increased after 2 and 4 h of exposure to TNF-α plus IL-17 as compared to that after exposure to TNF-α alone (Fig. 5i and k). Complementary to these findings, the nuclear localization of AUF-1 was observed in resting cells and it was about 2.5-fold higher after 2 and 4 h in TNF-α-stimulated cells compared to those exposed to TNF-α plus IL-17 (Fig. 5j and l). Both nuclear and cytoplasmic TTP (Supporting Information Fig. S5a and b) and KHSRP (Supporting Information Fig. S5d and e) were observed in lower amounts in resting cells, which increased after exposure to TNF-α for 2 h. However, there was no marked difference in TTP (Supporting Information Fig. S5c) and KHSRP (Supporting Information Fig. S5f) localization in cells exposed to TNF-α plus IL-17 as compared to that of TNF-α alone. Taken together, these findings strongly indicate that IL-17 limits miR16 involvement and regulates the binding of AUF-1 to IL-8 mRNA by translocation of AUF-1 from the nucleus to the cytoplasm.

### IL-17 modulates IL-6 and G-CSF expression also via miR16 and AUBps, but VEGF via miR16 only

**Figure 6:**
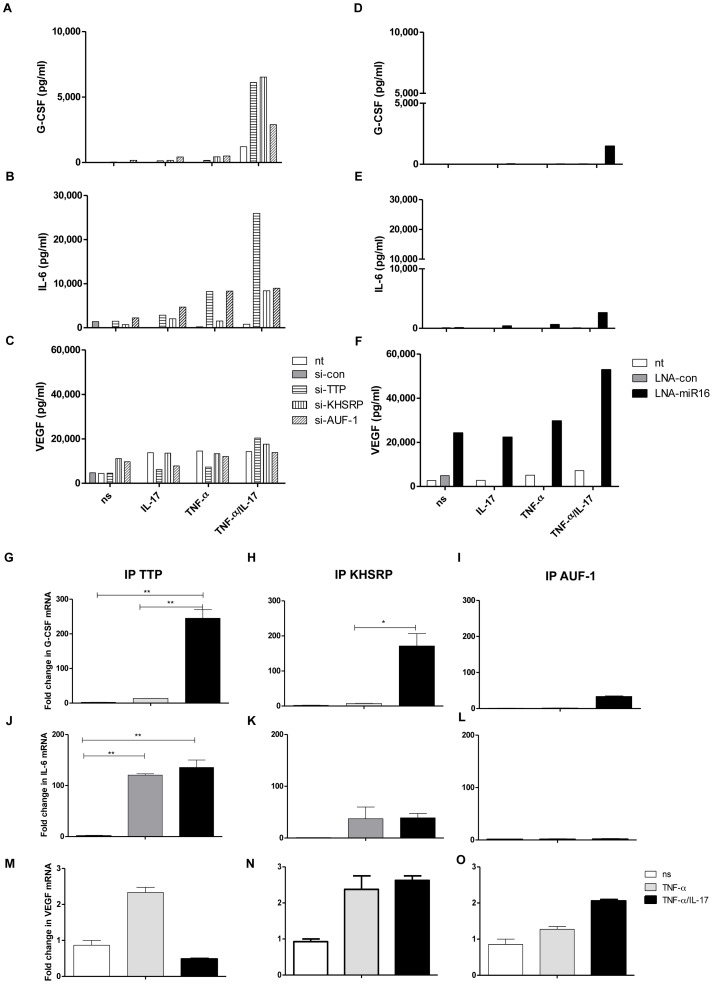
Involvement of AUBps and miR16 on expression of other inflammatory mediators and the effect of IL-17. **a–c**) Secreted levels of G-CSF (**a**), IL-6 (**b**) and VEGF (**c**) in supernatants of NCI-H292 cells, non-transfected (nt) or transfected with scrambled si-RNA (si-con), or si-TTP or si-KHSRP or si-AUF-1, unstimulated (ns) or stimulated with IL-17, TNF-α or TNF-α plus IL-17 for 24 h. **d–f**) Secreted levels of G-CSF (**d**), IL-6 (**e**) and VEGF (**f**) in supernatants of NCI-H292 cells, non-transfected or transfected with scrambled LNA (LNA-con), or LNA-directed against miR16 (LNA-miR16), unstimulated or stimulated with IL-17, TNF-α or TNF-α plus IL-17 for 24 h. Supernatants in duplicates from 3 experiments were mixed in equal amounts for the assay. **g–i**) Q-PCR showing fold change in G-CSF mRNA associated with immuno-purified TTP (**g**), KHSRP (**h**) and AUF-1 (**i**) in lysates from NCI-H292 cells stimulated for 2 h with TNF-α or TNF-α plus IL-17 with respect to non-stimulated (ns) cells. **j**–**l**) as g, h, i but now for IL-6 mRNA. **m–o**) as g, h and i but now for VEGF mRNA. ***P<0.001, ** P<0.01, * P<0.05(1-way ANOVA, Bonferroni's multiple comparison post-test). Representative results are shown for 3 experiments.

To determine whether the principles for the regulation of IL-8 expression and the effect of IL-17 also apply to other ARE-containing mRNAs we analyzed the expression of G-CSF, IL-6 and VEGF. G-CSF protein expression due to TNF-α plus IL-17 was attenuated by TTP and KHSRP, and less so by AUF-1 (Fig. 6a). In line herewith, G-CSF mRNA co-immuno-purified with TTP, KHSRP and to a lesser extent with AUF-1 (Fig. 6g, h and i). IL-6 protein expression was attenuated by TTP and less so by KHSRP and AUF-1 (Fig. 6b). Similarly, IL-6 mRNA showed strongest associations with TTP and less so with KHSRP and AUF-1 (Fig. 6j, k, and l). IL-17 plus TNF-α induced markedly higher levels of IL-6 (protein) as compared to TNF-α alone, which relates to TTP and KHSRP. However, the association of IL-6 mRNA to TTP (Fig. 6j) and KHSRP (Fig. 6k) is quite comparable for cells exposed to TNF-α or IL-17 plus TNF-α. This may relate to the fact that IL-6 mRNA in the immune-complexes was measured at 2 h after stimulation of the cells, whereas the proteins in the supernatant of the cells were measured after 24 h. In other words, it may well be that IL-6 mRNA bound to TTP and KHSRP in cells exposed to TNF-α is reduced at e.g. 4 h but not for cells exposed to IL-17 plus TNF-α. Anti-miR16 only affected G-CSF and IL-6 protein expression in cells exposed to TNF-α plus IL-17, although the effect was small in comparison to knocking down AUBps (Fig. 6d and e). Nevertheless, it appears that IL-17 modulates miR16 usage in AUBp-miR16 mediated regulation of gene expression. In contrast to IL-8, IL-6 and G-CSF mRNA regulation, VEGF mRNA was not regulated by any of the three AUBps studied here, as reflected by an unchanged VEGF protein expression despite knockdown of these AUBps (Fig. 6c). In accordance, no difference was observed in the degradation of VEGF mRNA in resting, TNF-α- or TNF-α plus IL-17-exposed cells in which AUBps were downregulated (Supporting Information Fig. S6a). Also, VEGF mRNA showed poor association with the immuno-purified AUBps (Fig. 6m, n and o). This suggests that TNF-α plus IL-17-induced regulation of ARE-containing mRNAs by AUBps is specific, but not all ARE-containing mRNAs are regulated by AUBps. Interestingly, inhibition of miR16 led to markedly exaggerated production of VEGF in cells exposed to TNF-α plus IL-17 but also, although to a lesser extent, at the other conditions (Fig. 6f). In addition, inhibition of miR16 led to attenuated VEGF mRNA degradation in cells exposed to TNF-α plus IL-17 and to a lesser extent to those exposed to TNF-α alone as compared to non-transfected cells (Supporting Information Fig. S6b). Together, this suggests that miR16 regulates VEGF expression independent of the AUBp-miR16 pathway. Collectively, these findings show that modulation of AUBp-miR16 mediated regulation of gene expression by IL-17 depends on the specific mRNA target, limiting miR16 usage and differential participation of the AUBps.

## Discussion

**Figure 7:**
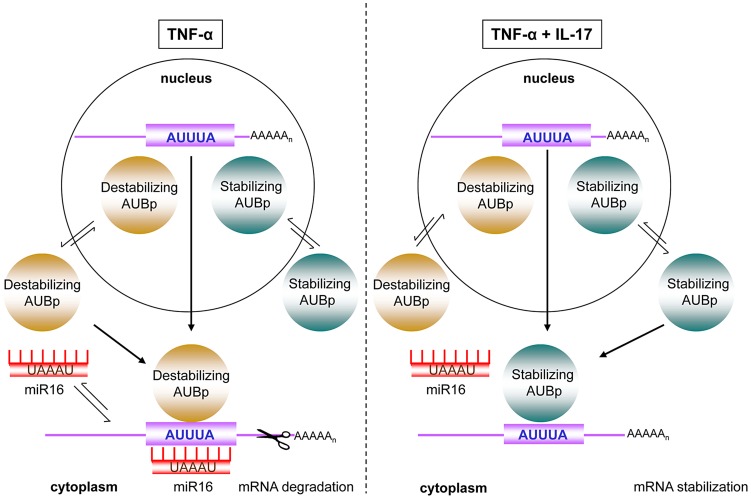
Model of IL-17 mediated regulation of ARE- mRNA expression by AUBps and miR16. Upon exposure to TNF-α, mRNAs of inflammatory mediators, containing ARE- sequences in the 3′UTR (ARE-mRNA), associate with destabilizing AUBps and miR16. The overall effect is ARE-mRNA degradation. In presence of TNF-α plus IL-17, miR16 association to ARE-mRNAs is limited and additional loading of stabilizing AUBps on the ARE-mRNA takes place, leading to attenuated ARE-mRNA degradation resulting in enhanced and prolonged expression of the encoded protein.

IL-17 is associated with chronic inflammatory diseases [3] and is increasingly being linked to the severity of inflammatory diseases such as in asthma [31]. This association likely arises from the synergy between IL-17 and pro-inflammatory stimuli like TNF-α in the production of pro-inflammatory mediators, particularly by stromal cells. For epithelial and fibroblast cells, IL-17 in combination with TNF-α has been shown to predominantly attenuate degradation of mRNAs encoding inflammatory mediators [9], [32], which results in a hyper-responsive production of these pro-inflammatory mediators [9], [33], [34]. Here we report that degradation of mRNAs encoding IL-8, IL-6 and G-CSF is directed by the AMD mRNA decay pathway, which depends on the concerted action of various AUBps and, interestingly, miR16. The relative contribution of each of the AUBps studied here and that of miR16 depends on the target mRNA. Degradation of IL-8 mRNA depended particularly on the AUBps TTP and KHSRP, and miR16, whereas IL-8 mRNA stabilization depended on AUF-1. IL-17 in presence of TNF-α changed the relative contribution of the various AUBps, best exemplified by the 400-fold enhanced binding of AUF-1 to IL-8 mRNA, protecting IL-8 mRNA from degradation. IL-17 in combination with TNF-α also attenuated expression of miR16, paralleled by reduced amounts of miR16 bound to the various immuno-purified AUBps, thereby dissipating the contribution of miR16 to IL-8 mRNA degradation. The relative contributions of these AUBps and that of miR16 were different in the cases of IL-6 and G-CSF but, as with IL-8 expression, IL-17 in presence of TNF-α also changed the relative contribution of these AUBps and that of miR16. This cooperation between miR16 and various AU-containing mRNAs, and the impact of the synergistic effect of IL-17 and TNF-α, has been depicted in Fig. 7. It is likely that other AUBps not studied here, such as HuR, TIA-1 and TIAR contribute to the expression of ARE-containing mRNAs in a manner similar to TTP, KHSRP and AUF-1. The role of TIA-1 and TIAR may be particularly interesting, as they have been implicated in translational control. Along similar lines other microRNAs, particularly those that contain complementary AU-rich-sequences similar to those of miR16, may fulfil a similar role as miR16.

In view of these findings it was unexpected that VEGF, which is encoded by a mRNA that also contains AU-rich elements in its 3′-UTR, was not regulated by any of the AUBps studied here. As yet, however, we cannot exclude the involvement of other AUBps. VEGF expression was controlled by miR16 at all conditions tested and most dependent in cells exposed to TNF-α plus IL-17 (Fig. 6f), which may relate to the IL-17-reduced miR16 levels in these cells. Interestingly, VEGF mRNA contains the seed sequence for miR16, but is not clear whether that determines the apparent independency of AUBps. Datta and colleagues [35] have found that the IL-17 regulated stability of CXCL1 mRNA was also independent of the existing AU-rich elements. In a follow-up study by this group [36] it was shown that IL-17 stabilized CXCL1 mRNA and several other mRNAs via TRAF5 and the splicing regulatory factor, SF2, which binds to 3′-UTR of CXCL1 mRNA, analogous to that reported for AUBps to the ARE sequences in the 3′-UTR of mRNAs. These findings likely relate to the notion that not all AU-rich elements are functional [37].

Jing and colleagues [30] have proposed that miR16 assists TTP in binding to its target TNF-α mRNA, possibly by the complementary sequences between miR16 and the AU-rich elements in TNF-α mRNA. Our data indicate that mRNA and miR16 indeed can complex with TTP, or KHSRP instead, but this association varies with conditions to which cells were exposed (unstimulated *versus* TNF-α *versus* TNF-α plus IL-17). In the presence of TNF-α plus IL-17, TTP and KHSRP were associated with target mRNAs in the absence of miR16. AUF-1 did not co-purify with miR16 at any of the conditions. So, it appears that miR16 is not required for binding of AUBps and their target mRNA. Its correlation with mRNA decay, however, suggests that miR16 may promote ARE-mediated mRNA degradation, in particular by KHSRP and TTP. IL-17 induced inhibition of miR16 expression as well as inhibition of miR16 using complementary LNA, resulted in an attenuated mRNA degradation. We have attempted to enhance miR16 expression by stable transfection and transduction with lentiviral constructs expressing miR16 (collaboration with InteRNA, Utrecht, The Netherlands) to determine whether that would promote mRNA degradation. Whereas other miR's were over-expressed by transduction with lentiviral constructs, we have not been able to enhance miR16 levels by stable transfection and transduction with lentiviral constructs expressing miR16, suggesting that miR16 expression may be strictly controlled. Our data also show that with reduced amounts of miR16 the stabilizing AUF-1 can outcompete TTP and KHSRP for binding to IL-8 mRNA and halt its degradation. The absence of a seed sequence for miR16 in IL-8 mRNA and other mRNAs indicate that miR16 may indeed interact by complementarity to the AU-rich elements, but other modes of interaction such as suggested recently [38] are not excluded.

Our methodology for purifying these complexes was aimed at preventing non-specific interactions, by meticulously eluting the AUBps using aspecific antibodies as well as a high and low salt elution steps. We have started to analyze the proteins within the complex where the presence of poly-A tail-binding protein (PABP) and ribosomal proteins (L5, S8, S19) suggest that this complex is close to that which promotes translation. This is supported by the observed correlations between the amount of target mRNA and the translated protein. As yet we have not found proteins that are part of the RISC complex.

Overall our data indicate that the interactions between AUBps, miR16 and the target mRNA largely depends on the specific RNA sequences and probably structural restraints. IL-17 exerts its function by changing the contribution of miR16 and AUBps. Besides IL-17, IL-1β has also been shown to attenuate mRNA degradation [9], [39]. As IL-17 did not synergize with IL-1β-induced IL-8 and IL-6 production by airway epithelial cells [9], it is likely that IL-1β uses the same mode of regulation as IL-17 [39].

The link between the AMD pathway and IL-17 is intriguing. The AMD pathway regulates the expression of 8% of the human transcriptome, comprising mRNAs encoding proteins that regulate a range of cellular and physiological processes. This implies that IL-17 may contribute to the pathophysiology of inflammatory diseases not only by enhancing the production of inflammatory mediators, but also by promoting the production of other regulatory proteins, which may affect processes such as tissue repair and cell cycling. If so, this underlines the relevance of targeting IL-17 in inflammatory conditions.

## Materials and Methods

### Cell culture and transient transfection and mRNA decay

The human lung mucoepidermoid carcinoma derived cell line NCI-H292 (CRL 1848; American Type Culture Collection [ATCC], Manassas VA) was cultured and propagated as described elsewhere [40]. NCI-H292 cells at 6×10^5^/ml were plated on 12-well plates (500 µl) and were grown till 70% confluence. Human (h)TTP si-RNA (sc-36760), hAUF-1 si-RNA (sc-37028), hKHSRP si-RNA (sc-44831), control si-RNA (si-con) (Fluorescein Conjugate) (sc-36869) were purchased from Santa Cruz Biotechnologies and hsa-miR-16 miRCURY LNA inhibitor probe, was purchased from Exiqon. Cells were transfected with si-KHSRP, si-TTP, si-AUF-1 or LNA-miR16 in presence of Lipofectamine 2000 transfection reagent (Invitrogen) according to manufacturer's instructions, at a final concentration of 50 nM in serum-free medium (Opti-MEM, Invitrogen). FAM-labeled scrambled si-RNA (si-con) and scrambled LNA (LNA-con) were used at a final concentration of 30 nM as controls for si-RNA and LNA-mediated knockdown, respectively, and to determine transfection efficiency. After transfection, cells were left to recover for 48 h followed by stimulation with recombinant human (rh) TNF-α (5 ng/ml) (R&D systems), rhIL-17 (100 ng/ml) (R&D systems), rhTNF-α plus rhIL-17 (5 ng/ml and 100 ng/ml, respectively) or no stimulus for 24 h for the assessment of protein production and for 2 h for the assessment of RNA. To study mRNA decay, RNA was isolated from cells at 0, 0.5 and 1 h after addition of Actinomycin D (ActD; 5 µg/ml, Sigma) using Trizol reagent (Invitrogen).

### Determination of inflammatory mediators

Cultured supernatants were assayed for IL-6 and IL-8 production, quantified by sandwich ELISA as described previously [34], [40] or, together with other inflammatory mediators, by multiplex assays (BioRad) and read in a Bioplex (BioRad).

### mRNA analysis

Total RNA was extracted from the cells with Trizol according to manufacturer's instructions and cDNA was synthesized with Revert Aid H Minus Reverse Transcriptase (Fermentas). Quantitative PCR reactions were done with Power Sybr green PCR master mix (Applied Biosystems) and run on Step One Plus Real Time PCR (Applied Biosystems). Details of primers are provided in the Supporting Information Table S1.

Expression of the gene of interest was first normalized to GAPDH control and is presented as normalized expression of the sample relative to the appropriate comparable condition (noted in the legend). A relative quantification was used to analyse the changes in gene expression in a given sample to that of a reference sample, an untreated control in this case. In immuno-purified samples, the expression of the gene of interest was determined from the respective standard curves and the fold change was calculated with respect to appropriate comparable condition (noted in the legend).

### miR 16 analysis

miR16 was analyzed using stem-loop primer (see Supporting Information Table S1) and quantitative PCR as described elsewhere [41]. Briefly, total RNA was extracted from the cells with Trizol according to manufacturer's instructions and cDNA was synthesized with Revert Aid H Minus Reverse Transcriptase (Fermentas) using miR16-1 (Stem loop) primer. Quantitative PCR reactions were done with Power Sybr green PCR master mix (Applied Biosystems) and run on Step One Plus Real Time PCR (Applied Biosystems).

### Immuno-purification

NCI-H292 cells (10×10^6^) were plated in T75 (15 ml) flasks and grown overnight. The cells were then left unstimulated or stimulated with TNF-α or TNF-α plus IL-17 for 2 h, after which cells were harvested and cytoplasmic lysates were prepared on ice as described before[42]. The lysates were pre-incubated with an isotype control antibody (Rb-anti-human-α2 macroglobulin, Dako A0033) and magnetic beads coated with protein A for 2 h at 4°C. μMACs protein A microbeads and MACs separation columns were purchased from Miltenyi Biotech.

The cell lysate was then loaded on MACs separation columns and the eluate was incubated with the desired antibody (KHSRP, TTP, AUF-1 or isotype control antibody) overnight at 4°C. Rabbit polyclonal anti-TTP (ab 33058), rabbit polyclonal anti-KHSRP (ab 960635) and rabbit polyclonal anti-hnRNP/AUF-1 (ab 61193) were purchased from Abcam. Goat polyclonal anti-β-actin and tubulin was purchased from Santa Cruz Biotechnologies. The lysates were re-loaded on separate columns for protein and RNA extraction. The columns were washed 5 times with low salt buffer (10 mM Hepes, 50 mM KCl, 100 mM NaCl, 5 mM MgCl_2_) and 3 times with high salt buffer (10 mM Hepes, 50 mM KCl, 500 mM NaCl, 5 mM MgCl_2_, 1% (v/v) NP40). The protein was eluted from the column with denaturing buffer (0.0625M Tris-HCl, pH 6.8, 2% SDS, 5% 2-mercaptoethanol, 10% glycerol, 0.002% bromophenol blue) heated to 90°C. RNA was eluted using 300 µl cold urea lysis buffer (7M urea, 2% (w/v) SDS, 0.35M NaCl, 10 mM EDTA, 10 mM Tris-HCl). RNA was extracted using equal volumes of Trizol according to manufacturer's instruction.

### Western blot

NCI-H292 cells were grown overnight and then left unstimulated or stimulated with TNF-α or TNF-α plus IL-17 for 2 h, after which cells were harvested and cytoplasmic and nuclear lysates were prepared on ice as described before[42]. Total protein in each fraction was measured by BCA protein assay kit (Thermo Scientific). Equal amounts of protein were electrophoresed on 13% SDS-PAGE on Bio-Rad Mini Protean II and Hoefer SE600 systems and transferred to PVDF membrane (Millipore). Blots were blocked with 0.4% (w/v) skimmed milk powder in phosphate-buffered saline and incubated with primary antibodies in the ratio Rb-anti AUF-1(1∶500) or Rb-anti-TTP (1∶100) or Rb-anti-KHSRP (1∶30) along with Goat anti-β-actin (1∶50) or Goat anti-tubulin (1∶50) and incubated at 4°C overnight followed by 1 h incubation at room temperature with IR dye-anti-Rabbit or anti-Goat secondary antibodies (1∶15000) (LI-COR Biosciences). The membranes were scanned and quantified using the Odyssey Infrared Imaging System (Li-COR Biosciences).

### Immunohistochemistry

NCI-H292 cells were seeded overnight at 5,000 cells in 200 µl per well in chambered slides (Lab-Tek, Thermo Fisher Scientific Inc.). The cells were stimulated with or without TNF-α or TNF-α plus IL-17 for 1, 2 and 4 h. The cells were then fixed with 4% formaldehyde (Merck) in PBS for 10 min, permeabilized with 4% formaldehyde and 0.1% Tween-20 (Merck) for 10 min and blocked with donkey serum (Santa Cruz Biotechnologies) in the same ratio as the respective primary antibodies for 1 h. Primary antibodies were then added in the ratio AUF-1: 1∶500; TTP: 1∶100 and KHSRP 1∶30 and incubated at 4°C overnight followed by 1 h incubation with fluorescent tagged secondary antibody (1∶400), Alexa Fluor goat anti-Rabbit 647, red, (A21245 Life Technologies) for AUF-1, TTP and KHSRP. Nuclei were counterstained with DAPI (Invitrogen). The samples were then analyzed with Leica Confocal Microscope SP-8 X SMD using LAS AF Lite software.

### Statistical analysis

The software GraphPad Prism 5 was used for statistical analyses (one-way and two-way ANOVA). *P* values of less than 0.05 were considered statistically significant.

## Supporting Information

Figure S1 RNA electro-mobility shift assay (REMSA). Cytoplasmic extracts were obtained from NCI-H292 cells after 2 h of stimulation with TNF-α or TNF-α plus IL-17 as described elsewhere [42]. The cell extract was incubated on ice in buffer containing Hepes 10 mM, pH 7.9, KCl 25 mM, NP40 0.05%, BSA 1 mg/ml, glycerol 5%, 1 mM DTT with ^32^P-labeled oligonucleotides, (Supporting Information Table S1) for 1 h and separated at 4°C on a 6% non-reducing PAGE at low voltage (30 V). Slow-migrating bands were obtained by using 1 µg of rabbit polyclonal anti-TTP. The data is representative of three experiments.
(PDF)

Figure S2 Competition assay with cold oligos for binding IL-8 mRNA. Cytoplasmic lysates from NCI-H292 cells stimulated with TNF-α or TNF-α plus IL-17 for 2 h were incubated on ice with ^32^P-labeled and unlabeled IL-8 mRNA probes in the ratio 1∶0, 1∶1, 1∶10 and 1∶50 for 1 h and separated at 4°C on a 6% non-reducing PAGE at low voltage. Data is representative of 2 experiments.
(PDF)

Figure S3 Western blots of serial fractions in immuno-purification of AUBps. Blots for KHSRP (**a**), TTP (**b**) and AUF-1 (**c**), showing the cytoplasmic lysate (Lys), pre-cleared lysate (PCL), flow through (FT), low salt wash (W1), high salt wash (W) and eluted proteins (E) from un-stimulated NCI-H292 cells as described in methods. Lane denoted M shows molecular weight markers. Data is representative of 3 experiments.
(PDF)

Figure S4 Association of IL-8 mRNA and miR16 in material purified by isotype control IgG. Fold change in IL-8 mRNA (**a**) and miR16 (**b**) associated with immuno-purified isotype control IgG in NCI-H292 cells stimulated for 2 h with TNF-α or TNF-α plus IL-17 as compared to non-stimulated (ns) cells.
(PDF)

Figure S5 Localization of TTP and KHSRP. Confocal images of cytoplasmic and nuclear localization of TTP (**a, b, c**) and KHSRP (**d, e, f**) in resting cells or cells stimulated with TNF-α and IL-17 plus TNF-α for 2 h. The nucleus is stained with DAPI. Data is representative of 4 experiments.
(PDF)

Figure S6 VEGF mRNA degradation. Non-transfected (ns) or si-con or si-KHSRP or si-TTP or si-AUF-1 (**a**) or LNA against miR16 (**b**) transfected cells were stimulated with TNF-α or TNF-α plus IL-17 for 2 h. RNA was isolated from cells at 0, 30 and 60 minutes (mins) after blocking gene transcription by actinomycin D (5 µg/ml). % of VEGF mRNA remaining was calculated on basis of q-PCR.
(PDF)

Table S1 List of oligonucleotides used in the experiments.
(PDF)
