# Supplementary figures and images for: IL-17 Attenuates Degradation of ARE-mRNAs by Changing the Cooperation between AU-Binding Proteins and microRNA16
Source: PLoS Genet. 2013 Sep 26;9(9):e1003747. doi: 10.1371/journal.pgen.1003747 (PMC3784493; doi:10.1371/journal.pgen.1003747)

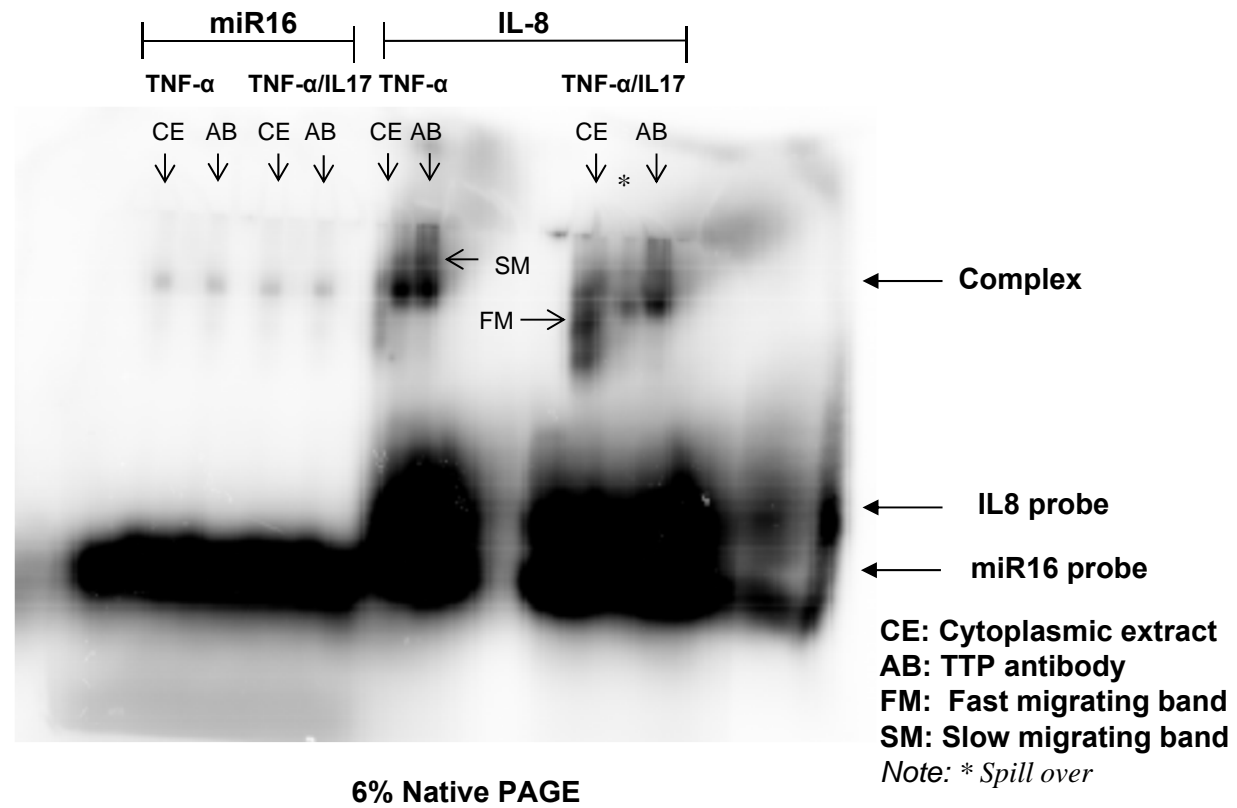

**Figure S1**

Supplement: Figure S1 — RNA electro-mobility shift assay (REMSA). Cytoplasmic extracts were obtained from NCI-H292 cells after 2 h of stimulation with TNF-α or TNF-α plus IL-17 as described elsewhere [42]. The cell extract was incubated on ice in buffer containing Hepes 10 mM, pH 7.9, KCl 25 mM, NP40 0.05%, BSA 1 mg/ml, glycerol 5%, 1 mM DTT with 32P-labeled oligonucleotides, (Supporting Information Table S1) for 1 h and separated at 4°C on a 6% non-reducing PAGE at low voltage (30 V). Slow-migrating bands were obtained by using 1 µg of rabbit polyclonal anti-TTP. The data is representative of three experiments. (PDF) [file pgen.1003747.s001.pdf]

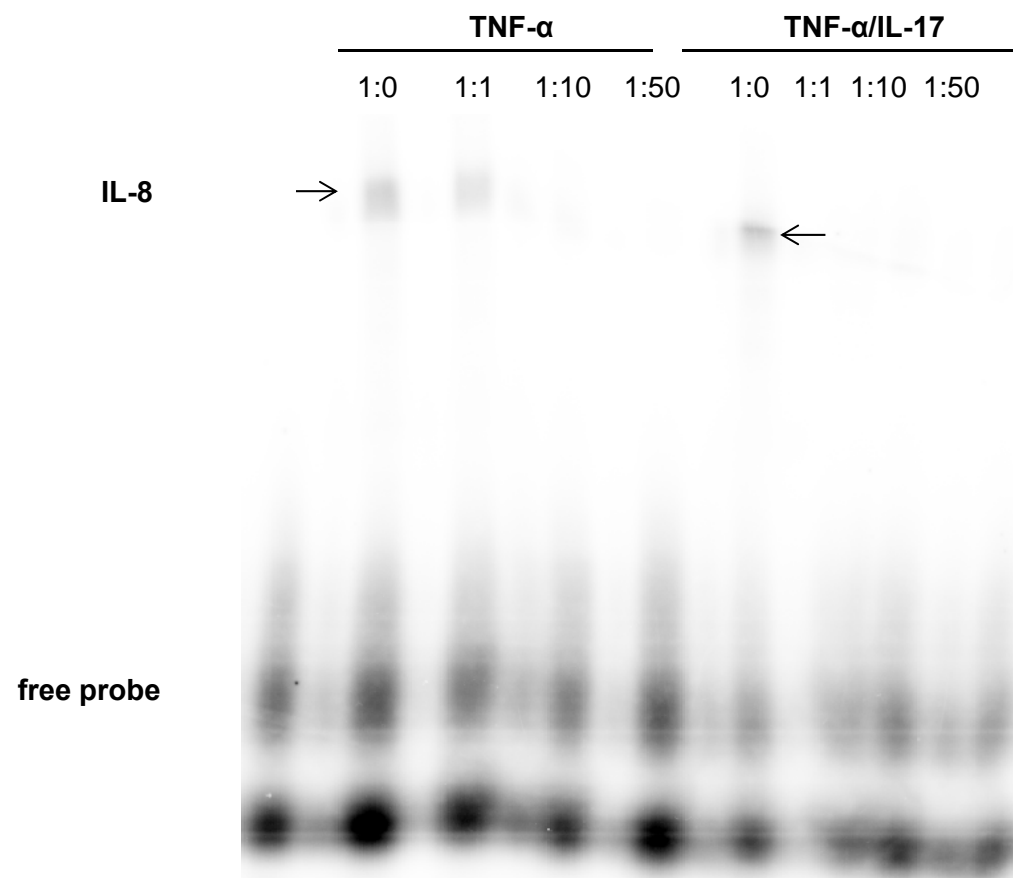

Figure S2

Supplement: Figure S2 — Competition assay with cold oligos for binding IL-8 mRNA. Cytoplasmic lysates from NCI-H292 cells stimulated with TNF-α or TNF-α plus IL-17 for 2 h were incubated on ice with 32P-labeled and unlabeled IL-8 mRNA probes in the ratio 1∶0, 1∶1, 1∶10 and 1∶50 for 1 h and separated at 4°C on a 6% non-reducing PAGE at low voltage. Data is representative of 2 experiments. (PDF) [file pgen.1003747.s002.pdf]

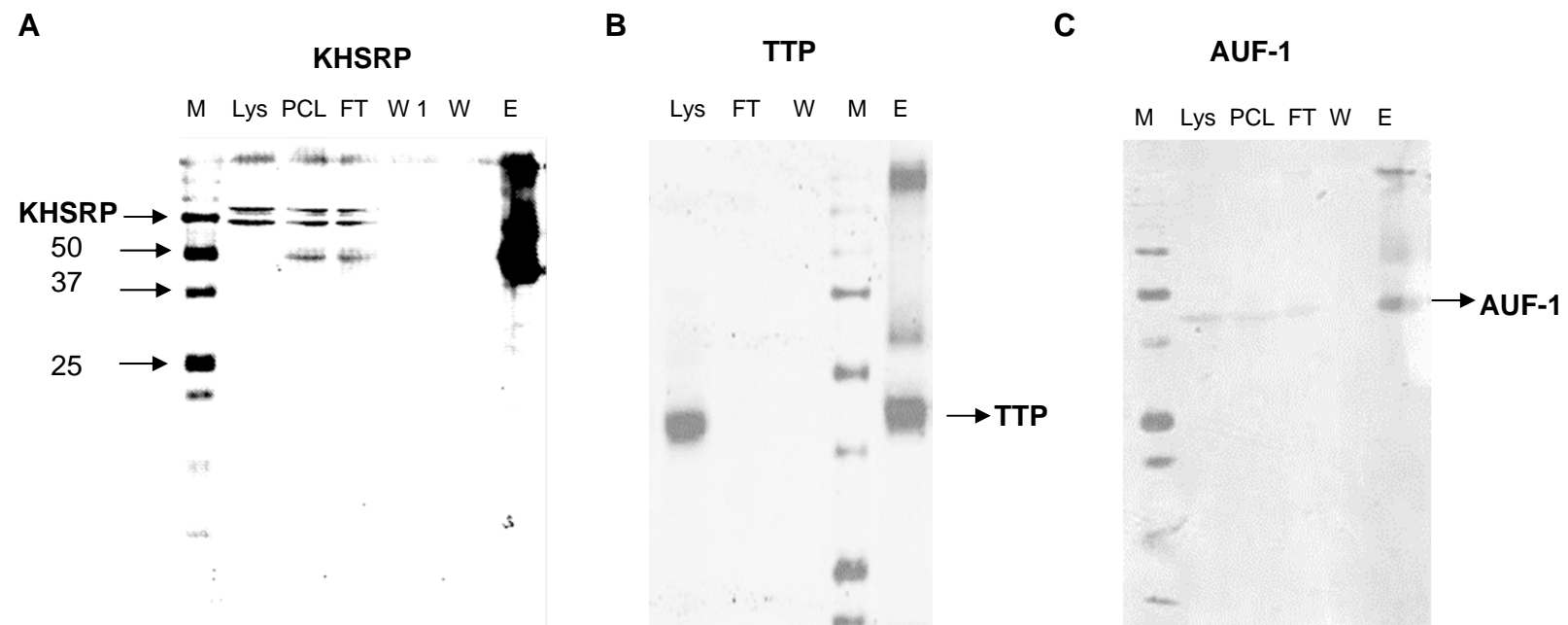

**Figure S3**

Supplement: Figure S3 — Western blots of serial fractions in immuno-purification of AUBps. Blots for KHSRP (a), TTP (b) and AUF-1 (c), showing the cytoplasmic lysate (Lys), pre-cleared lysate (PCL), flow through (FT), low salt wash (W1), high salt wash (W) and eluted proteins (E) from un-stimulated NCI-H292 cells as described in methods. Lane denoted M shows molecular weight markers. Data is representative of 3 experiments. (PDF) [file pgen.1003747.s003.pdf]

**A**

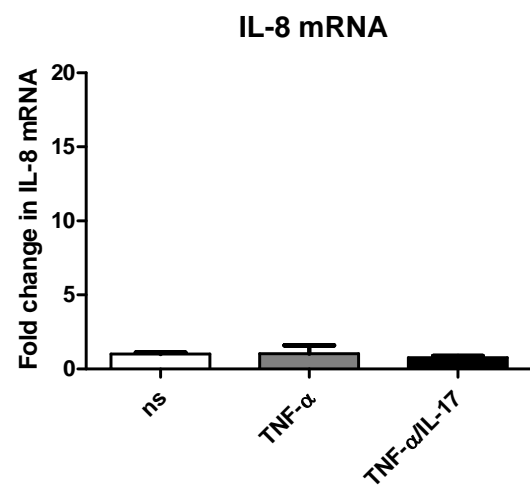

**B**

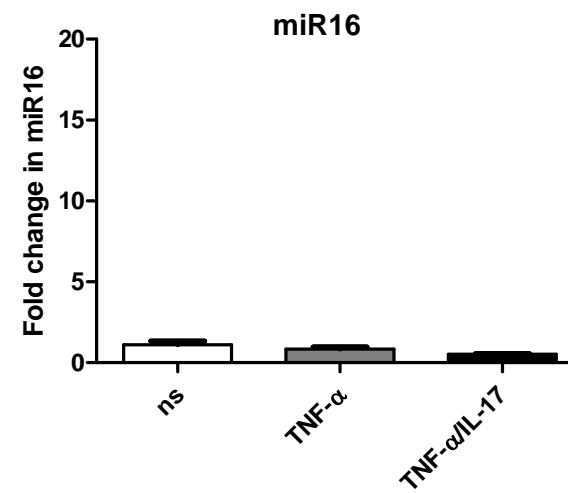

**Figure S4**

Supplement: Figure S4 — Association of IL-8 mRNA and miR16 in material purified by isotype control IgG. Fold change in IL-8 mRNA (a) and miR16 (b) associated with immuno-purified isotype control IgG in NCI-H292 cells stimulated for 2 h with TNF-α or TNF-α plus IL-17 as compared to non-stimulated (ns) cells. (PDF) [file pgen.1003747.s004.pdf]

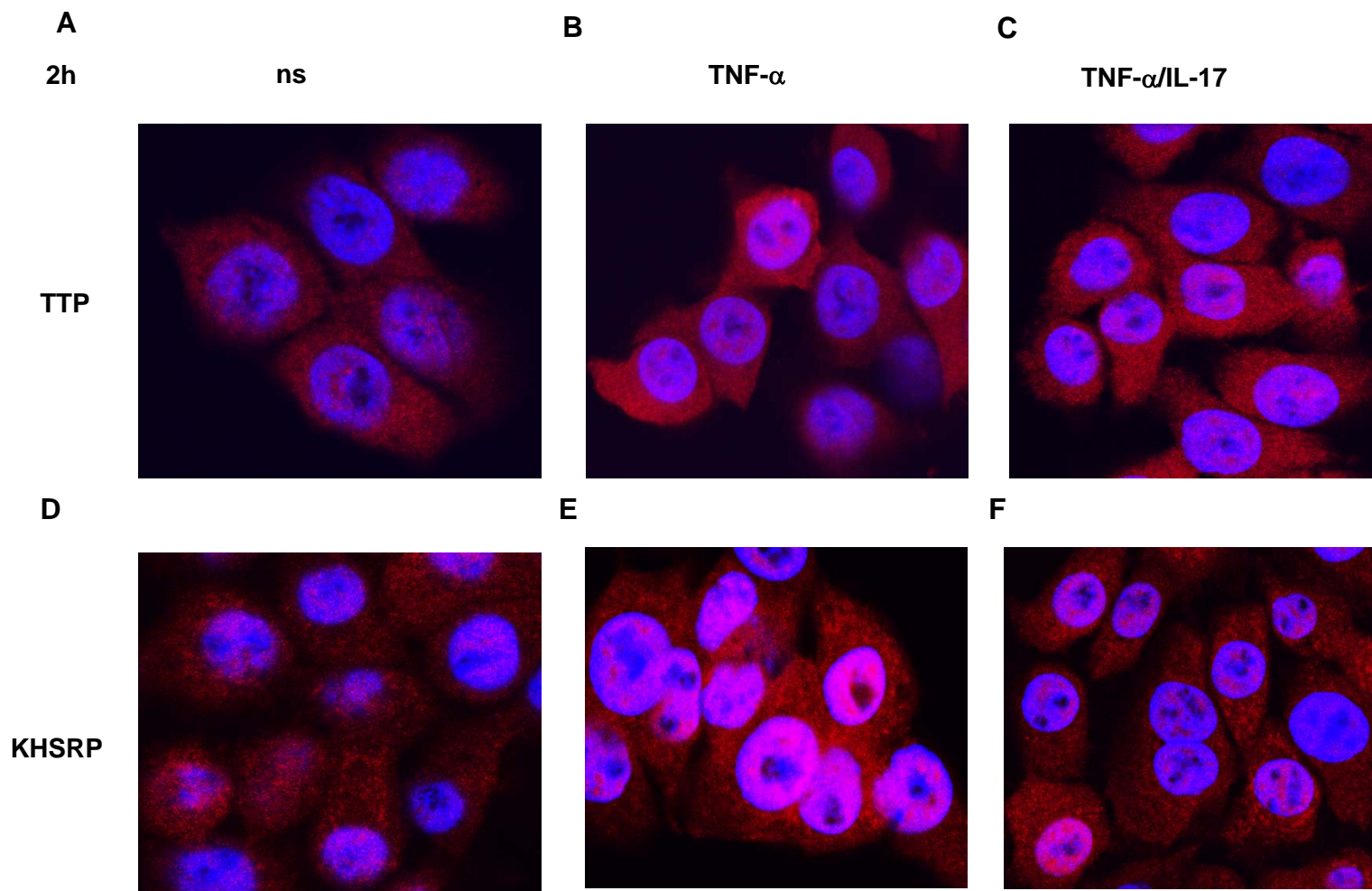

**Figure S5**

Supplement: Figure S5 — Localization of TTP and KHSRP. Confocal images of cytoplasmic and nuclear localization of TTP (a, b, c) and KHSRP (d, e, f) in resting cells or cells stimulated with TNF-α and IL-17 plus TNF-α for 2 h. The nucleus is stained with DAPI. Data is representative of 4 experiments. (PDF) [file pgen.1003747.s005.pdf]

**A**

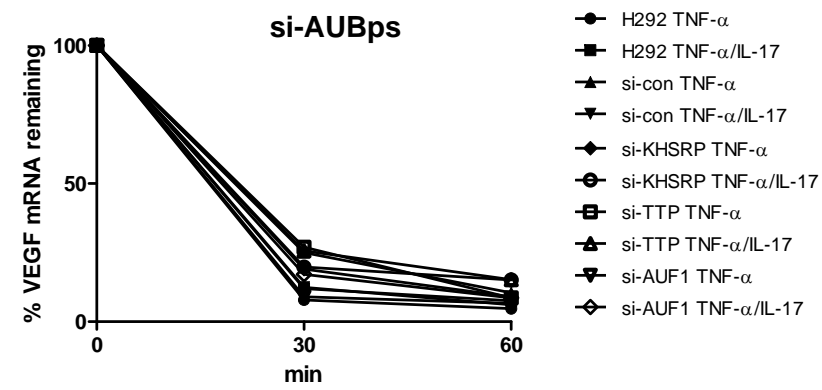

**B**

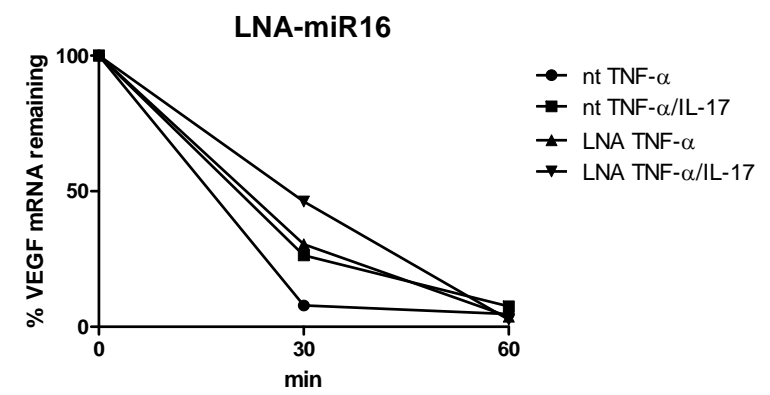

**Figure S6**

Supplement: Figure S6 — VEGF mRNA degradation. Non-transfected (ns) or si-con or si-KHSRP or si-TTP or si-AUF-1 (a) or LNA against miR16 (b) transfected cells were stimulated with TNF-α or TNF-α plus IL-17 for 2 h. RNA was isolated from cells at 0, 30 and 60 minutes (mins) after blocking gene transcription by actinomycin D (5 µg/ml). % of VEGF mRNA remaining was calculated on basis of q-PCR. (PDF) [file pgen.1003747.s006.pdf]
