# Supplementary material for: IL-17 Attenuates Degradation of ARE-mRNAs by Changing the Cooperation between AU-Binding Proteins and microRNA16
Source: PLoS Genet. 2013 Sep 26;9(9):e1003747. doi: 10.1371/journal.pgen.1003747 (PMC3784493; doi:10.1371/journal.pgen.1003747)
Supplement: Table S1 — List of oligonucleotides used in the experiments. (PDF) [file pgen.1003747.s007.pdf]

Analysis	Oligonucleotides used
mRNA analysis	Hu IL-8 PPH00568A, SA Biosciences<br>Hu IL-6 PPH00560B, SA Biosciences<br>Hu CSF3 PPH00723A, SA Biosciences<br>Hu VEGFA PPH00251B, SA Biosciences<br>Hu GAPDH F : TCA TCT CTG CCC CCT CTG C<br>Hu GAPDH R : GAG TCC TTC CAG GAT ACC AA
miR 16 analysis	MiR16-1 (Stem loop) GTC GTA TCC AGT GCA GGG TCC GAG GTA TTC GCA CTG GAT ACG ACC GCC AAT AT<br>MiR 16-1 (Forward) TGC GGT AGC AGC ACG TAA AT<br>MiR16-1 (Reverse) TGC AGG GTC CGA GGT AT
EMSA	hsa-miR-16 miRCURY ™ LNA detection probe, Exiqon<br>IL-8 AAC TTC TCC ACA ACC CTC TG

Table S1
